# Folate status in Germany

**DOI:** 10.17886/RKI-GBE-2016-040.2

**Published:** 2016-12-14

**Authors:** Gert B.M. Mensink, Anke Weißenborn, Almut Richter

**Affiliations:** 1 Robert Koch Institute, Department for Epidemiology and Health Monitoring, Berlin, Germany; 2 Federal Institute for Risk Assessment, Department of Food Safety, Berlin, Germany

**Keywords:** NUTRITION, FOLATE, HEALTH SURVEY, DEGS1, GERMANY

## Abstract

Folate is important for cell division and thus for growth and physical development. Serum folate concentrations can be used to assess a population’s folate status. In Germany, the median serum folate level of adults aged 18 to 79 is 7.5 ng/ml (women: 7.9 ng/ml, and men: 7.2 ng/ml). Approximately 86% of the adult population in Germany has adequate folate levels. Higher folate concentrations are observed among older age groups and individuals with a higher socio-economic status. However, the World Health Organization recommends that women of reproductive age should, at the population level, have significantly higher folate levels in order to reduce the risk for neural tube defects. However, the majority of women in this age group do not achieve these concentrations.

## Introduction

Folate refers to the naturally occurring form of this water-soluble B vitamin. Many types of fruit and vegetables (such as green leafy vegetables, cucumbers and tomatoes) as well as potatoes, meat, and bread and pastries made from wholegrain flour are good sources of folate. However, a significant amount of folate can be lost during food preparation, e.g. through exposure to heat [1]. The synthetic form of folate – folic acid – is used in dietary supplements and fortified foods. The two forms are chemically distinguishable: folic acid is more stable when exposed to light, heat and oxygen, and has a higher level of bioavailability than folate [1, 2]. Folate is required for a number of important body functions including cell growth, division and differentiation. An adequate supply of folate, therefore, is particularly important during pregnancy as it can significantly reduce the risk for embryonic malformations (neural tube defects), foetal growth retardation, premature birth, miscarriage and low birth weight [3]. There is also discussion about a possible contribution of folate in the prevention of cardiovascular diseases, neurodegenerative diseases and cancer. However, there is insufficient evidence to support a preventive impact of folate supply beyond general requirements [1].

A population’s folate status may be estimated in different ways: firstly, information on folate intake via foods that people consume can be collected (using data gathered through consumption surveys); secondly, serum and erythrocyte folate concentrations can be measured. Whereas erythrocyte folate is a good indicator of an individual’s long-term folate status, serum folate levels provide information about an individual’s current folate status. Although serum folate concentrations are subject to individual and daily variations, they are still considered an appropriate biomarker for assessing the population’s folate status [1, 6]. In the following, results are presented of serum folate analyses collected in the ‘German Health Interview and Examination Survey for Adults’ (DEGS1), which was conducted by the Robert Koch Institute between 2008 and 2011 [4, 5].

## Indicator

As part of DEGS1, serum folate concentrations were analysed for 7,045 individuals (3,669 women and 3,376 men) aged 18 to 79 years using a chemiluminescent microparticle immunoassay (CMIA, Architect system, Abbott Wiesbaden). A serum folate concentration ≥ 4.4 ng/ml is assumed to be adequate for adults. In contrast, concentrations < 3 ng/ml indicate clinical folate deficiency [1, 6]. In the following, the serum folate concentrations are presented in relation to gender, age and socio-economic status.

## Reflection of the results

The median serum folate level measured in DEGS1 was 7.5 ng/ml overall, with 7.9 ng/ml for women and 7.2 ng/ml for men (Table 1). This means that the median value for women and men is significantly higher than the reference level of ≥ 4.4 ng/ml (representing an adequate folate status for adults). Similarly, the German National Health Interview and Examination Survey 1998 (GNHIES98), which was conducted between 1997 and 1999 by the Robert Koch Institute, recorded a median serum folate concentration among its 18- to 40-year-old female participants of 7.6 ng/ml [7]. Therefore, on average, women’s folate levels seem to have remained relatively unchanged since the late 1990s (GNHIES98 did not evaluate men’s serum folate levels).

DEGS1 demonstrated a positive correlation between serum folate concentrations and age among women and men: in 18- to 29-year-olds, the median serum folate concentration was 6.9 ng/ml for women and 6.3 ng/ml for men, while in the 70- to 79-year-old age group, the concentration was 8.7 and 8.2 ng/ml for women and men, respectively (Table 1). DEGS1 also showed a correlation between serum folate levels and socio-economic status, particularly among women (and less strongly among men) (Table 1). These findings can probably be explained by the fact that older age groups and people with a higher socio-economic status are more likely to eat more fruit and vegetables [8].

Approximately 86% of the adult population (around 88% of women and 84% of men) has adequate serum folate concentrations of ≥ 4.4 ng/ml (Table 1). The percentage increases with age. Approximately 12% of women and 16% of men have serum folate values < 4.4 ng/ml, indicating an inadequate folate status. The prevalence of people with an inadequate folate status is significantly higher among 18- to 29-year-olds (about 17% of women and about 21% of men) than in the 70- to 79-year-old age group (about 11% of women and about 9% of men). A total of 3.3% of adults (2.5% of women and 4.1% of men) have serum folate levels below 3 ng/ml, indicating clinical folate deficiency (Table 1). Again, the percentage of people with clinical folate deficiency is higher among younger aged groups (18- to 29-year-olds: 3.3% of women and 5.8% of men; 70- to 79-year-olds: 2.3% of women and 0.6% of men).

In summary, serum folate concentrations compared with the reference value demonstrates that approximately 86% of the adult population in Germany have an adequate folate status. However, it is important to consider that the reference value applied is intended for the folate status of the general population. For women of reproductive age, however, a higher reference value has been suggested in order to reduce the risk for embryonic neural tube defects. Consequently, WHO recommends that, on a population level, women of reproductive age should have an erythrocyte folate concentration of at least 400 ng/ml [9]. If this recommendation is applied to the biomarker of erythrocyte folate, which has also been measured in DEGS1, 95% of the women would not have adequate levels of folate [10]. A similar situation was already identified in GNHIES98 among women of reproductive age [7]. The present results thus emphasize the importance of the recommendation for periconceptional folic acid supplementation [1], which is followed insufficiently in Germany. It has, however, to be considered that neural tube defects are caused by numerous factors. It is therefore impossible to predict the individual risk of neural tube defects based solely on the WHO-reference value for folate levels in erythrocytes. Folate measurements as presented here, thus, merely serve to provide as an estimate of the folate status of the female population of reproductive age.

In addition to biomarkers, the folate supply can be estimated by use of food intake data gathered via consumption surveys. The German Nutrition Society (DGE) recommends an adult intake of 300 μg folate equivalents per day (equivalents take into account the different bioavailability of folate and folic acid). This rises to 450 μg/day when breastfeeding and to 550 μg/day during pregnancy. Clearly, an adequate consumption of folate-containing foods is required to prevent folate deficiency. Alongside a folate-rich diet, however, the DGE recommends that women who wish to have a child should take a daily supplement containing 400 μg folic acid in order to reduce the risk of neural tube defects. They should start supplementation at least 4 weeks before pregnancy and maintain it during the first trimester of pregnancy [1]. The National Food Consumption Survey II, which was carried out between 2005 and 2006 by the Max Rubner-Institut, observed that women and men aged between 14 and 80 had a median folate intake of 184 μg/day and 207 μg/day, respectively [11]. Thus, the majority of the adult population failed to meet the reference values for folate intake. Nevertheless, it is important to note that the reference values are intended to assure that at least 97.5% of the population has adequate folate concentrations [1]. Therefore, intake reference values also contain safety margins for people with increased folate needs. Therefore, an assessment of the overall folate supply can also be based on the estimated average requirement (EAR), which indicates the average daily level of intake estimated to meet the needs of 50% of individuals. A study that compared various EU countries, using an EAR of 150 μg/day, revealed that the folate intake of the population in Germany is comparatively adequate [12].

## Key statements

- Approximately 86% of the adult population in Germany have adequate folate levels.
- Folate status improves with increasing age.
- Most women of reproductive age do not meet the World Health Organization’s recommended population level for red blood cell folate to reduce the risk for neural tube defects.
- Individuals with a higher socio-economic status generally have higher folate levels.

## Figures and Tables

**Table 1:** Serum folate among 18- to 79-year-olds according to gender, age and socio-economic status Source: DEGS1 (2008–2011)

	n	Median	Serum folate	
		ng/ml	< 3 ng/ml	≥ 4.4 ng/ml
**Women (total)**	**3,669**	**7.9**	**2.5%**	**88.2%**
**Age**				
18 – 29 years	539	6.9	3.3%	82.9%
30 – 39 years	429	7.4	2.6%	85.6%
40 – 49 years	691	7.6	2.5%	87.7%
50 – 59 years	752	8.3	2.1%	90.4%
60 – 69 years	712	9.0	2.1%	94.5%
70 – 79 years	546	8.7	2.3%	89.3%
**Socio-economic status**				
Low	595	7.3	4.3%	81.6%
Medium	2,280	7.9	2.4%	88.9%
High	771	8.5	1.1%	94.2%
**Men (total)**	**3,376**	**7.2**	**4.1%**	**84.1%**
**Age**				
18 – 29 years	515	6.3	5.8%	79.1%
30 – 39 years	406	6.7	4.4%	80.3%
40 – 49 years	597	7.1	5.1%	81.3%
50 – 59 years	640	7.5	4.8%	86.2%
60 – 69 years	668	7.9	1.7%	91.1%
70 – 79 years	550	8.2	0.6%	91.0%
**Socio-economic status**				
Low	518	6.7	7.2%	78.8%
Medium	1,934	7.3	3.7%	84.0%
High	900	7.4	2.7%	89.6%
